# Premature Ejaculatory Dysfunction in Rheumatoid Arthritis (PED-RA Study)

**DOI:** 10.12669/pjms.38.8.5698

**Published:** 2022

**Authors:** Abrar Ahmed Wagan, Sultan Ahmed Chandio, Paras Surahyo

**Affiliations:** 1 Dr. Abrar Ahmed Wagan, MBBS, FCPS (Medicine), FCPS (Rheumatology), FACR. Associate Professor of Rheumatology, Indus Medical College, Tando Mohammad Khan; 2 Dr. Sultan Ahmed Chandio, MBBS, FCPS (Medicine) Assistant Professor of Medicine SMBBMU, Larkana, Pakistan; 3 Dr. Paras Surahyo, MBBS, FCPS (Radiology) Senior Registrar, Bilawal Medical College, Jamshoro, Pakistan

**Keywords:** Sexual dysfunction, Premature ejaculation, Rheumatoid Arthritis, DAS-28

## Abstract

**Objective:**

To determine the frequency of premature ejaculatory dysfunction in rheumatoid arthritis patients.

**Methods:**

After approval from IRB, cross sectional study was conducted from November 1^st^, 2020 to August 1^st^, 2021 at Department of Rheumatology, Indus Medical College, Pakistan. RA patients were included, written and informed consents were taken. Demographic data was noted and detailed history and examination was carried out. Each participant BMI and Blood pressure was measured. Afterward 5-ml of blood was drawn by a trained phlebotomist for CBC, ESR, fasting blood sugar levels, HBs Ag and anti HCV Antibody test.DAS-28 Calculator was used for RA clinical activity. Premature ejaculation diagnostic tool was used for the assessment of premature ejaculation.

**Results:**

Total 168 patients with mean age 32.27 (SD=±9.49) and mean disease duration of 6.35 (SD=±3.95) years were included,. Prevalence of premature ejaculation dysfunction was (44.6%), with mean PED of score 9.17 (SD=±5.23). Hypertension, HCV, Hakeem medications, use of DMARDS had positive association while High BMI and higher DAS-28 has negative association with PED (p<0.05).

**Conclusion:**

There is high prevalence of PED in RA, it needs proper evaluation, treatment and urgent research is needed to know more about it.

## INTRODUCTION

Rheumatoid arthritis (RA) is a chronic inflammatory joint disease that affects 0.5–1% of population worldwide. It’s an erosive arthritis if left uncontrolled and untreated results into articular damage, multiple comorbids, disability and reduced quality of life.^1^ Sexuality is considered an important and integral part of quality of life and responsible for our individual wellbeing. Sexual dysfunction (SD) is defined as a change in any component of sexual activity, which may cause frustration, pain, leading to decreased sexual intercourse. Auto-immune diseases like RA affects the quality of sexual life, but sexual dysfunction is still underdiagnosed and underreported in such patients, due to two major factors: (i) shame or frustration from subject (ii) doctors doesn’t pay attention to this aspect of disease. Act of sex consists of the following phases: (1) Desire: characterized by fantasies about sexual activity. (2) Excitation: subjective feeling of sexual pleasure along with physiological changes; (man) penile tumescence, (woman): pelvic vascular congestion, lubrication, vaginal expansion, and swelling of the external genitalia, (3) Orgasm: climax of sexual pleasure, with release of sexual tension and rhythmic contraction of perineal muscles and reproductive organs (in man); sensation of ejaculatory inevitability, followed by ejaculation, (4) Resolution: feeling of relaxation and general well-being.^2^

Overall, the prevalence of sexual dysfunction (SD) ranges from 18.4% to 30%) in men, and (25.8% to 67%) in women. major male Sexual dysfunctions includes erectile dysfunction (ED), diminished libido, and abnormal ejaculation, while the four major categories of female sexual dysfunctions are desire, arousal, orgasmic, and sexual pain disorders these problems are multifactorial affected by biological, psychological, and interpersonal determinants.^3^ RA most common in females patients, they often experience a decrease in sexual desire, difficulties in sexual positions, and a lower rate of orgasms, due to RA related clinical symptoms (pain, fatigue, limitations of joint mobility and morning stiffness).^4^-^8^ Along with classical RA clinical features other potential mechanisms of RA and male SD shared similar pathological factors as in females, such as pain, fatigue, joint mobility, depression, and Hypoandrogenicity.^9^-^12^ The proportion of male patients with RA reporting SD ranged across (37% to 66%).^13^,^14^ The accepted definition of Premature ejaculation Dysfunction (PED) comes from the DSM-IV-R and ICD-10, as a condition of short ejaculatory latency that causes personal distress and is beyond the patient’s ability to control with worldwide prevalence of 30%.^15^

Patients with chronic diseases don’t get time in clinics and because of socio-cultural environment/taboos, shy away from discussing about sex related issues, to make rheumatologists aware about the impact of RA on PED. Our objective was to determine the frequency of premature ejaculatory dysfunction in rheumatoid arthritis patients.

**Scatter Plot 1:**
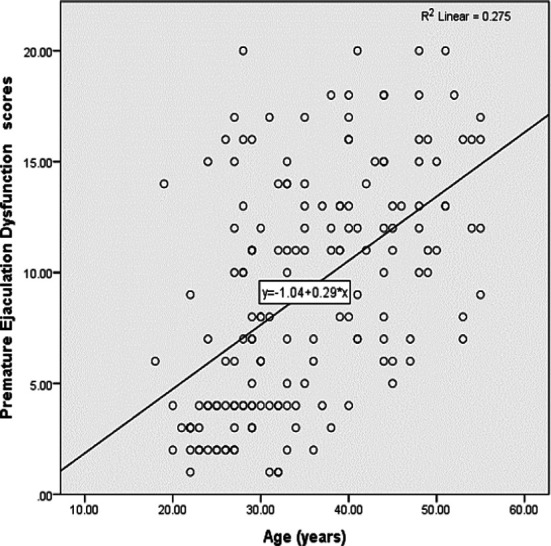
Association of PED Scores with Age Scatter plot -1 showing a positive correlation between age and PED scores, R2 showed there was 27.5% variation in PED was explained by age.

**Scatter Plot 2:**
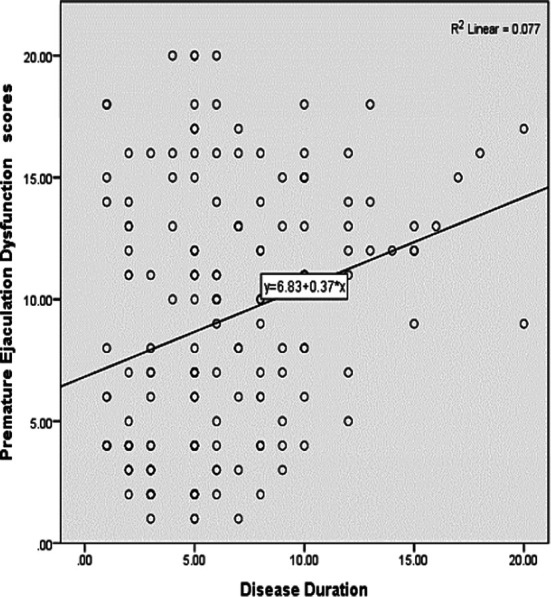
Association of PED Scores with Duration of Diseases. Scatter plot-2 showing a positive correlation between disease duration and PED scores, R2 showed there was 7.7% variation in PED was explained by disease duration.

## METHODS

After the approval of IRB (IMC/37/2020-1-10-2020) this cross-sectional study was done at Department of Rheumatology Indus Medical College Tando Mohammad Khan. Written and informed consent was taken from each study participant. A sample size of 168 cases was calculated.

Only Seropositive RA participants were taken. Sero-negative RA, SLE, Scleroderma, Sjogren syndrome, MCTD, psoriatic arthritis, primary osteoarthritis, primary or secondary fibromyalgia, polymyalgia rheumatic, primary impotence, peyronies disease, hypothyroidism, hypogonadism, recurrent UTI, history of chemotherapy or radiotherapy in last two years, use of biological and targeted synthetic DMARDs, known cases major depression or any psychiatric illness, history of pelvic surgery in last one year were excluded.

A pre-defined questionnaire was used to collect demographic data; contains information like; years since diagnosis, marital status, smoking habits. Each participant BMI and Blood pressure was measured as defined protocols. Afterward 5- ml of blood was drawn by a trained phlebotomist for CBC, ESR, fasting blood sugar levels, HBs Ag and anti HCV Antibody test.

Premature ejaculation diagnostic tool was used for the assessment of Premature ejaculation, out of total score of 20 if score was >11 participant was labeled as suffering from PE, while score of 9-10 labeled as probable and score of < 8 as negative or least possible PED.

### Statistical Analysis:

Data were stored and analyzed using IBM-SPSS version 23.0, mean with standard deviation reported for quantitative data sets, counts with percentages given for qualitative data sets. Frequency of Premature ejaculation dysfunction was calculated. Association was tested using Pearson Chi square test with studied parameters. Scatter plots were done to see the trend of PED scores with age and duration of disease. P-values less than 0.05 were considered statistically significant.

## RESULTS

The baseline characteristics,168 study participants, 92.3% were married, the mean age of samples was 32.27 (SD=±9.49) years, 32.1% were smokers,17.3% were diabetes mellitus, 26.8% were hypertensive, 11.3% were HCV, 8.9% were hepatitis surface antigen positive, 28.6% reported for Hakeem medication, the mean BMI of samples was 28.35 (SD=±5.57) kg/m2, 32.7% were normal weight BMI, the mean DAS-28 of samples was 3.46 (SD=±1.36), 26.8% found with moderate DAS-28, the mean duration of disease was 6.35 (SD=±3.95) years, and mean Premature Ejaculation dysfunction score was 9.17 (SD=±5.23). The frequency of Premature Ejaculation Dysfunction, PED confirmed (44.6%), probable PED (6.5%) and non-PED (48.6%). Table-I

**Table-I:** Baseline Characteristics of Studied Samples (n=168).

Characteristics		n	%
Marital Status	Unmarried	5	3.0
	Married	155	92.3
	Divorced / Widow but having active sexual activity	8	4.8
Age (years)	Mean ±SD	35.27	±9.49
Smoking	Yes	54	32.1
	No	114	67.9
DM	Yes	29	17.3
Hypertension	Yes	45	26.8
HCV	Yes	19	11.3
HBS	Yes	15	8.9
Hakem Medication	Yes	48	28.6
BMI levels	Normal	55	32.7
	Underweight	58	34.5
	Obese	55	32.7
	Mean ±SD	28.34	±5.57
DAS-28	Remission	47	28.0
	Mild	49	29.2
	Moderate	45	26.8
	High Disease Activity	27	16.1
	Mean ±SD	3.46	±1.36
Disease Duration (years)	Mean ±SD	6.35	±3.95
Premature Ejaculation Dysfunction scores	Mean ±SD	9.17	±5.23

The association of PED with studied factors, among PED samples there were 42.7% samples underweight BMI, 90.7% were married, 38.7% were smokers, 21.3% were DM, 41.3% were hypertensive, 21.3% were Hepatitis C virus antibody positive, 9.3% hepatitis B antigen positive, 46.7% uses Hakeem medication, 70.7% were on single of multiple DMARDS, 34.7% were mild DAS-28. Pearson Chi Square test gives as significant association of BMI, Hypertension, HCV, Hakeem medication, DMARDS and DAS-28 with PED, (p<0.05).Table-II

**Table-II:** Association of Premature Ejaculation Dysfunction with Studied Parameters

Parameters		Premature Ejaculation Dysfunction						

		Yes (n=75)		NO (n=82)		Probable (n=11)		p-value

		n	%	n	%	n	%	
BMI levels	Normal	26	34.7	23	28.0	6	54.5	0.03*
	Underweight	32	42.7	24	29.3	2	18.2	
	Obese	17	22.7	35	42.7	3	27.3	
Marital Status	Unmarried	1	1.3	4	4.9	0	0.0	0.26
	Married	68	90.7	76	92.7	11	100.0	
	Divorced / Widow but having active sexual activity	6	8.0	2	2.4	0	0.0	
Smoking	Yes	29	38.7	21	25.6	4	36.4	0.20
DM	Yes	16	21.3	11	13.4	2	18.2	0.42
Hypertension	Yes	31	41.3	11	13.4	3	27.3	<0.01*
HCV	Yes	16	21.3	2	2.4	1	9.1	<0.01*
HBS Ag	Yes	7	9.3	8	9.8	0	0.0	0.55
Hakeem medication	Yes	35	46.7	8	9.8	5	45.5	<0.01*
DMARDS	No Medication ever used	22	29.3	4	4.9	2	18.2	<0.01*
	Single drug Methotrexate	13	17.3	38	46.3	3	27.3	
	Methotrexate plus Hydroxychloroquin	15	20.0	30	36.6	4	36.4	
	Methotrextae +Hydroxychloroquin + sulphasalazine	15	20.0	4	4.9	1	9.1	
	Methothrexate+HCQ+ SSZ+Leflunomide	1	1.3	4	4.9	1	9.1	
	Used anyone of them are all but left treatment	9	12.0	2	2.4	0	0.0	
DAS-28	Remission	28	37.3	18	22.0	1	9.1	<0.01*
	Mild	26	34.7	20	24.4	3	27.3	
	Moderate	9	12.0	31	37.8	5	45.5	
	High Disease Activity	12	16.0	13	15.9	2	18.2	

* p<0.05 was considered statistically significant using Pearson Chi Square test.

## DISCUSSION

If RA is left untreated there is vicious circle of Pain-physical disability-psychological issues, ultimately leading to poor quality of life. Sexuality, its expression, and completion of act, is vital for healthy and diseased persons, as it’s a crucial aspect of an individual’s self-identity and self-esteem.^10^

Perez-Garciaa LF et al in his very recently published meta-analysis reported that RA not only affects the sexual functions but also impairs the quality of semen and sperm count, and disease activity has profound affect over the sexual functions, similarly we found that those who had higher disease activity had more premature ejaculation problem DAS-28 (0.05).^16^

In a Romanian study, with cohort of 60 patients, having mean age was 45.26 (7.8) years with various autoimmune rheumatic disease (rheumatoid arthritis (RA) - 17%, cases), 21,66% of the patients reported erectile dysfunction (ED) in comparison with only 8,33% in the control group (p=0.009) in comparison our cohort was very young in age.^17^ Danish study results showed some alarming statistics that; majority of patients with RA (93.5% of women and 85.5% of men) had not discussed sexual issues with a health-care professional during the last five years. Of all, 32.5% wished that their health care provider must discuss about sexual health.^18^. Systematic review and metaanalysis results demonstrated that RA was significantly associated with an increased risk of SD in females (RR 1.73, 95% CI 1.36-2.22, p < 0.001; heterogeneity: I2 60.3%, p = 0.028) as well as in males (RR 1.99, 95% CI 1.64-2.43, p < 0.001) and suggests that both patients and clinicians should be aware of the potential role of RA in the development of SD.^19^ A Pakistani study has, reported 13% prevalence of premature ejaculation and it was reported to be the second most common sexual dysfunction in Non RA population.^20^ In comparison our study PED has prevalence of (44.6%), and (6.5%) were probable PED. Madhukar et al, had reported that, in rheumatoid arthritis out of the 50 patients, 40 had some kind of sexual disability / dysfunction, while, six out of 40 participants had erectile dysfunction, 11 out of 25 reported reduced libido, out of which two blamed chronic medication as the culprit, two out 40 admitted to altered body image to be the reason for sexual dysfunction and 35 out of 40 patients complained of pain/stiffness of joints/functional limitations/ fatigue as the prime cause.^21^

In real practice sexuality and sexual problems remains a restricted area of discussion for patients and physicians for various reasons, an area that many feel unable to discuss. Frequent reasons provided by healthcare professionals are poor training or education in sexual health, lack of experience, religious or personal views, belief that the topic is not important or appropriate, and embarrassment on part of patients to open up with doctor about sexual health.^22^,^23^

Although exact cause of premature ejaculatory dysfunction couldn’t be ascertained in general and RA patients however there are various factors related to it, Corona et al found that many men with premature ejaculation have, low serum prolactin levels.^24^ In RA there is wide prevalence of depression and dyslipidemia, diabetes mellitus, obesity, illicit drug use, so these might be affecting the sexual functions as well.^25^-^27^

Majority of medications used for arthritis do not impairs sexual functioning, but some cases of erectile impotence and peyronie’s disease have been reported with methotrexate, sulfasalazine, and hydroxychloroquine, other drugs like (cimetidine, diclofenac, misoprostol, and naproxen) may interfere with libido, loss of desire, while difficulty with orgasm are common side effects of antidepressant medications. Yet results are still far from unequivocal.^28^-^30^

### Limitations:

Sample is from one center, number of participants is small, so results can’t be generalized, to best of our knowledge this is first study of its kind, which has focused on one entity of sexual dysfunctions called premature ejaculatory dysfunction, and this may pave the way for future studies on this aspect in local population.

## CONCLUSION

Rheumatoid arthritis is not limited to joints only, rheumatologists have to think beyond this, premature ejaculatory dysfunction along with other sexual dysfunctions is very common. Encourage patients to speak up about sex related issues in countries like Pakistan where it is considered as taboo to discuss.

### Authors’ Contribution:

**AAW:** Design, drafting, data acquisition, analysis, interpretation,. final approval

**SAC:** Data acquisition, data analysis, data interpretation, drafting, final approval,

**PS:** Data acquisition, data analysis, data interpretation, drafting, final approval.

All authors are accountable for integrity of the study.
